# The change in quality of life after Muller's muscle-conjunctival resection surgery for eyelid ptosis repair

**DOI:** 10.1007/s10792-025-03498-2

**Published:** 2025-03-30

**Authors:** Daniel David, Guy Ben Simon, Mordechai Rosner, Ayelet Priel, Oded Sagiv, Daphna Landau Prat, Ofira Zloto

**Affiliations:** 1 Sackler Faculty of Medicine, Tel Aviv University, Tel Aviv, Israel; 2 Goldschleger Eye Institute, Sheba Medical Center, Tel Hashomer, Israel

**Keywords:** Blepharoptosis, Eyelid ptosis, Muller’s muscle-conjunctival resection, Quality of life, Glasgow benefit inventory questionnaire

## Abstract

**Objective:**

To evaluate the long-term effect of the Muller's Muscle-Conjunctival Resection (MMCR) procedure on patient's quality of life.

**Methods:**

This retrospective study included patients that underwent MMCR surgery for eyelid ptosis repair at our institution between the years 2016–2018 and had at least 3 months postoperative follow up. All patients completed the 18-questions post-interventional, validated, Glasgow Benefit Inventory (GBI) questionnaire via telephone interview.

**Results:**

50 patients were included in the study group. The mean MRD1 measurement before ptosis repair surgery was 1.65 mm (± 0.82), with significantly higher MRD1 after a long post-operatively period, of 3.2 mm (± 1.03) (Matched pairs, *p* < *0.001*). The mean total GBI score was 57.48 (± 4.72) out of 90, after an average follow up period of 712 days.

**Conclusions:**

The MMCR eyelid ptosis repair surgery has a positive, long-term effect on patient’s quality of life. Good patient selection for specific type of ptosis surgery, based on pre-operative clinical characteristics, can result good outcome with high satisfaction rates.

**Supplementary Information:**

The online version contains supplementary material available at 10.1007/s10792-025-03498-2.

## Introduction

Blepharoptosis (ptosis) is a downward displacement of the upper eyelid margin [1]. Ptosis results from congenital, myogenic, involutional, neurogenic, mechanical or traumatic causes. [2, 3] The surgical technique for ptosis repair depends on the patient’s clinical characteristics and patient and surgeon preference and includes the Muller’s Muscle-Conjunctival Resection (MMCR), Levator Aponeurosis Advancement (LAA) surgery, Fasanella procedure, and the frontalis suspension procedure [3]. The indications for ptosis surgery are both functional and cosmetic. Some patients are concerned about a sleepy-looking appearance or that they look older than their actual age [4], while others complain about functional problems, such as superior visual field defects, a compensatory chin-up backward head tilt, difficulty in reading, and contrast sensitivity problems [5]. Those indications are discussed by physicians, patients and also by health care insurance companies when surgical repair is considered.

Quality of life after different types of surgeries has been investigated widely in the last decade [6]. Different scoring systems and questionnaires were used in order to measure the quality of life after surgical interventions. Recent studies in the field of oculoplastic have used the Glasgow Benefit Inventory (GBI) to assess the quality of life after different oculoplastic surgeries [7–10].

The improvement in the visual field and its association to quality of life after LAA is well documented [11–13]. Maycock et al.[9] showed an improvement in quality of life as assessed by high GBI score in patients that underwent LAA surgery. Mahroo et al.[10] demonstrated similar results in a study of 50 patients, most of them after LAA surgery and a single patient after MMCR. To our knowledge, most of the ptosis quality of life studies assessed the improvement after LAA and after a short time period. Therefore, this study aims to investigate the effect of the MMCR procedure on a patient’s quality of life, and to determine whether this effect is influenced by the patient’s demographics and clinical characteristics.

## Methods

Adult patients who underwent the MMCR procedure for ptosis repair surgery at the Goldschleger Eye Institute, Sheba medical center, and had a follow-up period of at least 3 months in a period between 2016 and 2018 were included. Patients with other ocular or orbital surgeries or with other eye problems were excluded.

In all patients, the operated eye was included in the statistical analysis. When both eyes were operated on, only the right eye was included for analysis. Separate analysis was performed for the left eye and resulted in similar outcomes.

The following data was retrieved for analysis: Age, gender, method of anesthesia, surgical and post-surgical complications, and whether a second ptosis repair surgery was required. Pre-operative and post-operative clinical features were also noted and included the best corrected visual acuity (BCVA), intra-ocular pressure (IOP), presence of dermatochalasis, corneal, anterior, and posterior chamber pathologies, measurements of Margin to Reflex Distance 1 (MRD1), Levator Function (LF) Palpebral Fissure (PF) and ephrin test result.

The primary outcome was the quality of life after ptosis repair surgery, as evaluated by the GBI questionnaire score. The secondary outcome was determining whether the quality of life was affected by the demographics or the patient's clinical features before and after surgery.

The study was approved by the institutional review board and adheres to the tenets of the Declaration of Helsinki.

### Glasgow benefit inventory (GBI)

The GBI is a validated, post-interventional questionnaire, first used in otorhinolaryngological studies [14]. It consists of 18 questions regarding the patient's quality of life, which can be subdivided into general, social, and physical aspects. Patients are asked to answer these questions on a 1 to 5 scale, one meaning a major deterioration in their quality of life and five being a major improvement. The full GBI questionnaire is presented in the Supplemental 1.

Patients included in the study were contacted via telephone call and supplied verbal consent to participate in the study and to answer the GBI questionnaire. After identification and patient consent, questions were read from the GBI form, and answers were documented.

### Statistical analysis

Quantitative variables were described as mean and standard deviation. Categorical variables were described as absolute and relative frequencies.

Matched pairs analysis was performed to evaluate the change after surgery compared to before surgery.

The overall significance level was set to an alpha of 0.05. The statistical analysis was carried out using Microsoft Excel 2017 (Microsoft Corporation, Redmond, WA) and IBM SPSS software version 24.0 (SPSS, Inc., Chicago, IL, USA).

## Results

A total of 50 patients (35 females, 15 males) were included in the study, of which 43 (86%) had bilateral eyelid surgery and 7 (14%) were operated on in only one eye. The mean (SD, range) age of participants was 68.36 (± 9.16, 48–84).

### Clinical characteristics

41 patients (82%) underwent concomitant blepharoplasty during their ptosis repair surgery. The majority of patients (49, 98%) were operated under local anesthesia, with a single patient that underwent general anesthesia. Other operatives, pre-operative and post-operative characteristics are presented in Table 1.

**Table 1:** Demographic, operative, pre-operative and post-operative characteristics of patients that underwent ptosis repair surgery

	N (%)	Mean	SD	Range
*Demographics*				
Age (years)	50 (100)	68.3	9.1	48–84
Gender (females)	35 (70)	–	–	–
*Operative characteristics*				
Concomitant blepharoplasty	41 (82)	–	–	–
*Anesthesia*				
Local	49 (98)	–	–	–
General	1 (2)	–	–	–
*Pre-operative characteristics*				
BCVA (logMAR)	47 (94)	0.22	0.19	0–1
MRD1 (mm)	47 (94)	1.65	0.82	0–3
LF (mm)	38 (76)	14.76	1.96	10–18
IOP (mmHg)	33 (66)	14	2.85	10–22
*Ephrin test*				
Positive	34 (68)	–	–	–
N/A	16 (32)	–	–	–
*Post-operative characteristics*				
BCVA (logMAR)	50 (100)	0.2	0.12	0–1.56
MRD1 (mm)	36 (72)	3.20	1.03	1–4
IOP (mmHg)	23 (46)	14	3.97	8–24

*IOP* intra-ocular pressure, *LF* levator function, *MRD* margin to reflex distance, *N/A* not available

The pre-operative clinical features such as corneal, anterior, and posterior segment pathologies were compared with the post-operative features, with no significant difference between them.

A positive ephrin test was noted in 34 patients before MMCR surgery, and the mean levator function was 14.7 mm.

### Prognosis

The mean MRD1 measurement before ptosis repair surgery was 1.65 mm (± 0.81) while post-operatively, it was significantly higher 3.2 mm (± 1.03) (Matched pairs, *p* < *0.001*). No significant correlation was found between the pre-operative MRD1 measurement and the GBI score (correlation coefficient = 0.108; T = 0.774).

No significant difference was noted when comparing IOP or BCVA pre and post operatively (Matched pairs, *0.582 and p* = *0.630*, respectively).

After a mean follow up time of 285 days (SD ± 324, range 31–1847), two patients (4%) had under-correction that required second ptosis repair surgery. No patient suffered from any other major surgical or post-surgical complications.

### Quality of life after surgery

Participants completed the GBI questionnaire at an average time of 712 days (SD ± 369, range 275–2108) after surgery.

All patients in the study answered the entire 18-questions GBI questionnaire with a mean (SD) score of 57.48 (± 4.54) out of 90. The highest score was given to the second question (4.16, ± 0.86) regarding the patient’s overall improvement in quality of life, and the lowest to the ninth question (2.66, ± 0.59) about the patient's feeling of self-consciousness after surgery. The mean GBI score for all the 18 questions are presented in Table 2.

**Table 2:** results of the 18-questions Glasgow Benefit Inventory (GBI) questionnaire

Question	Mean	SD	Range
Q1	3.96	0.903	1–5
Q2	4.16	0.866	1–5
Q3	3.54	0.908	2–5
Q4	3.04	0.493	1–4
Q5	3.22	0.648	2–5
Q6	3.14	0.405	3–5
Q7	2.92	0.396	2–4
Q8	3.06	0.470	1–5
Q9	2.66	0.593	1–4
Q10	3.00	0	3
Q11	3.94	0.314	1–3
Q12	2.88	0.328	2–3
Q13	4.02	0.845	2–5
Q14	3.06	0.314	3–5
Q15	3.06	0.314	3–5
Q16	2.90	0.364	2–4
Q17	2.92	0.274	2–3
Q18	3.00	0.202	2–4

*GBI* Glasgow Benefit Inventory, *Q* question, *SD* Standard Deviation

## Discussion

This study was designed to evaluate a patient's quality of life after MMCR surgery for eyelid ptosis repair. Previous studies have shown the objective, clinical advantages of ptosis repair surgery, as well as the subjective, self-reported, long-term benefit on a patient’s quality of life [11, 13]. This current study used a validated quality of life questionnaire, the Glasgow Benefit Inventory (GBI). Moreover, while previous studies focused on the LAA procedure [9, 10], our study group included only patients that underwent MMCR.

The high mean total GBI score measured in our study after ptosis surgery validates the positive effect of ptosis repair surgery on a patient’s quality of life. Furthermore, the relatively long follow up period (mean of 2 years post-surgery) suggests lasting patient’s satisfaction from this procedure. The highest score (mean of 4.16 on a 1–5 scale) was given to question number two, which asked if the patient’s overall life became better or worse after the surgery, reflecting our primary outcome as close as possible. Therefore, it seems that ptosis surgery improves not just patient’s visual field and appearance but also their quality of life, even after a long time period after surgery. This should be taken into account by surgeons and patients when considering the option of ptosis surgery repair.

Battu et al.[13] found an improvement in quality of life after LAA surgery. They evaluated quality of life by using a specific, self-designed, 27-item questionnaire. Maycock et al.[9] et al. also found an improvement in patient’s quality of life after LAA surgery, using the GBI questionnaire. This study focused on patients that underwent the MMCR procedure. MMCR surgery is considered a less complicated surgery compared to LAA that takes less time [15] and is as effective as LAA for relevant patients [16]. We found that quality of life of patients after MMCR surgery has improved significantly and remained so for a long period of time post-operatively.

One of the primary clinical outcomes after ptosis repair surgery, that is an increase in the MRD1 measurement, was significantly better after the operation relative to the measurements prior to surgery. Most patients that underwent MMCR had a positive ephrin test, while their levator function was normal, suggesting good patient selection for this specific type of procedure.

This study is not without any limitations, most of them derive from the fact that this is a retrospective study. The study group is relatively small and further research with a larger number of subjects would help strengthen the generalizability of these findings. Moreover, the quality of life of patients was not evaluated prior to surgeries. Further studies are warranted to evaluate the quality of life and clinical outcomes before MMCR compared to after MMCR.

In conclusion, this study presents new data regarding the positive effect of the MMCR procedure on a patient’s quality of life, as reflected by the high GBI score after surgery. Therefore, MMCR surgery for ptosis repair can improve a patient’s quality of life. Good patient selection based on pre-operative clinical characteristics may result in a good surgical outcome and high long term satisfaction rates. To the best of our knowledge this is the first study that evaluates quality of life after MMCR.

## Supplementary Information

Below is the link to the electronic supplementary material.

Supplementary file1 (DOCX 16 KB)

## Data Availability

No datasets were generated or analysed during the current study.
