# Supplementary material for: The change in quality of life after Muller's muscle-conjunctival resection surgery for eyelid ptosis repair
Source: Int Ophthalmol. 2025 Mar 30;45(1):130. doi: 10.1007/s10792-025-03498-2 (PMC11955430; doi:10.1007/s10792-025-03498-2)
Supplement: Supplementary file 1 — Supplementary file1 (DOCX 16 KB) [file 10792_2025_3498_MOESM1_ESM.docx]

| **Question** |
| --- |
| 1. Have the results of your operation affected the things you can do? |
| 2. Have the results of your operation made your overall life better or worse? |
| 3. Since your operation have you felt more or less optimistic about the future? |
| 4. Since your operation do you feel more or less embarrassed when with a group of people? |
| 5. Since your operation do you have more or less self-confidence? |
| 6. Since your operation have you found it easier or harder to deal with company? |
| 7. Since your operation have you been to your doctor, or any reason, more or less often? |
| 8. Since your operation do you feel more or less confident about job opportunities? |
| 9. Since your operation do you feel more or less self-conscious? |
| 10. Since your operation are there more or fewer people who really care about you? |
| 11. Since you had the operation, do you catch colds or infections more or less often? |
| 12. Since your operation have you had to take more or less medicine, for any reason? |
| 13. Since your operation do you feel better or worse about yourself? |
| 14. Since your operation do you feel you have more or less support from your family? |
| 15. Since your operation do you feel you have more or less support from your friends? |
| 16. Since your operation have you been more or less inconvenienced by your other health problems? |
| 17. Since your operation have you been able to participate in more or fewer social situations? |
| 18. Since your operation have you been more or less inclined to withdraw from social situations? |
|  |
| Questions were answered on a 5-point Likert scale:  1 = Much worse  2 = A little or somewhat worse  3 = No change  4 = A little or somewhat better  5 = Much better |

**Glasgow benefit inventory questionnaire**
